# Influenza A Virus-Driven Airway Inflammation may be Dissociated From Limb Muscle Atrophy in Cigarette Smoke-Exposed Mice

**DOI:** 10.3389/fphar.2022.859146

**Published:** 2022-03-18

**Authors:** Kevin Mou, Stanley M. H. Chan, Kurt Brassington, Aleksandar Dobric, Simone N. De Luca, Huei Jiunn Seow, Stavros Selemidis, Steven Bozinovski, Ross Vlahos

**Affiliations:** School of Health and Biomedical Sciences, RMIT University, Melbourne, VIC, Australia

**Keywords:** viral exacerbation, COPD, muscle weakness, fiber type transformation, lung-to-muscle axis, myogenic disruption, conditioned medium

## Abstract

Limb muscle dysfunction is a hallmark of Chronic Obstructive Pulmonary Disease (COPD) which is further worsened following a viral-induced acute exacerbation of COPD (AECOPD). An amplified airway inflammation underlies the aggravated respiratory symptoms seen during AECOPD, however, its contributory role to limb muscle dysfunction is unclear. The present study examined the impact of influenza A virus (IAV)-induced exacerbation on hind limb muscle parameters. Airway inflammation was established in male BALB/c mice by exposure to cigarette smoke (CS) for 8 weeks. Exacerbation was then induced via inoculation with IAV, and various lung and muscle parameters were assessed on day 3 (peak of airway inflammation) and day 10 (resolution phase) post-infection. IAV infection exacerbated CS-induced airway inflammation as evidenced by further increases in immune cell counts within bronchoalveolar lavage fluid. Despite no significant impact on muscle mass, IAV exacerbation worsened the force-generating capacity of the tibialis anterior (TA) muscle. Protein oxidation and myogenic disruption was observed in the TA following CS exposure, however, IAV exacerbation did not augment these detrimental processes. To further explore the contributory role of airway inflammation on myogenic signaling, cultured myotubes were exposed to conditioned medium (CM) derived from bronchial epithelial cells stimulated with polyinosinic:polycytidylic acid and cigarette smoke extract (CSE). Despite an amplified inflammatory response in the lung epithelial cells, the CM derived from these cells did not potentiate myogenic disruption in the C2C12 myotubes. In conclusion, our data suggest that certain parameters of limb muscle dysfunction seen during viral-induced AECOPD may be independent of airway inflammation.

## Introduction

Chronic obstructive pulmonary disease (COPD) is a heterogenous, incurable inflammatory disorder associated with exposure to noxious particles or gases, with the leading causative factor being cigarette smoking (CS) (Barnes, 2018). The disease is characterized by persistent airflow limitation, and the combination of small airway disease and parenchymal destruction that are progressive in nature (Decramer and Janssens, 2013). Recent studies have shown that COPD is a complex disease with both pulmonary and extrapulmonary manifestations (Vanfleteren et al., 2016). Skeletal muscle dysfunction is an important extrapulmonary comorbidity of COPD (Decramer and Janssens, 2013; Passey et al., 2016) and causes a rapid decline in the quality of life, and premature death (Gosselink et al., 1996; Marquis et al., 2002). People with COPD often experience acute deterioration of respiratory symptoms (e.g., cough, sputum production, and dyspnea), known as acute exacerbation of COPD (AECOPD) (Gosker et al., 2021). Although AECOPD may be caused by different etiological factors including those of a non-infectious nature, respiratory viruses are identified in 30–50% of all AECOPD episodes (Mathioudakis et al., 2020), in which 25% of these cases are primarily caused by influenza A virus (IAV) (Mackay and Hurst, 2013). In addition to the diagnostic respiratory symptoms, heightened pulmonary inflammation is a key pathological feature of viral AECOPD, driven by a combination of excessive proinflammatory cytokine/chemokine production (IL-6, IL-8, TNF-α, IFN-γ, GM-CSF), excessive oxidative stress, and protease-antiprotease imbalances (Barnes, 2017). The recruitment of inflammatory cells such as neutrophils, into the lungs further increases the secretion of proinflammatory factors, superoxide production, and protease release, resulting in the amplification of lung inflammation and destruction of airway structures (Barnes, 2018). Consequently, the presence of respiratory viruses during an exacerbation of COPD has been correlated to worsened symptoms and a delayed recovery (Mathioudakis et al., 2020).

The term “muscle dysfunction” encompasses the loss of force generating capacity (i.e., strength), loss of mass, and increase in fatigability of a muscle. These are commonly observed in the limb muscles of COPD patients (Vogelmeier et al., 2017) and of note, are predominantly seen in the lower limbs (Gosselink et al., 1996; Bernard et al., 1998). The preferential impact on the lower limbs may be related to a greater reduction in activity of these muscles in people with COPD (Maltais et al., 2014), together with the weight bearing nature of the lower limb muscles. During AECOPD, muscle strength, mass, and fatigability are found to be further impaired, which may lead to a longer duration of hospitalization and readmission within the following 3 months (Girón et al., 2009). Moreover, exacerbation-linked muscle dysfunction has been demonstrated to be an independent predictor of future hospitalization for COPD patients (Schols et al., 2005; Amado et al., 2019). Because of this, attempts have been made to retain/restore muscle mass in these patients with androgenic anabolic steroids. Despite the short-term benefits on lean body mass and muscle size, there were no consistent effects on muscle strength, exercise capacity, or pulmonary function (Pan et al., 2014). Moreover, long-term use of anabolic steroids was found to be associated with a number of adverse effect such as insulin resistance, cardiovascular disease, hepatotoxicity and hormonal imbalance effects (Pan et al., 2014). Therefore, it is of importance to understand the key mechanisms responsible for the further deterioration of muscle dysfunction in AECOPD.

A number of clinically relevant risk factors are believed to contribute to muscle dysfunction in AECOPD, including physical inactivity, malnutrition, therapeutic interventions (e.g., anti-inflammatory steroids) and the “spill-over” of airway inflammatory mediators into the systemic circulation (Barnes and Celli, 2009; Bernardo et al., 2015). It has been postulated that airway inflammation alone is sufficient to cause muscle dysfunction (Files et al., 2012), and furthermore, attenuation of airway inflammation by noninvasive mechanical ventilation reduces systemic inflammation during AECOPD (Hillas et al., 2016). This raises an important observation that airway inflammation may be directly linked to the further deterioration of muscle dysfunction seen in AECOPD, particularly when IAV infection has been shown to exacerbate airway inflammation (Bozinovski et al., 2012; Wang et al., 2019). The present study aimed to investigate the effect of IAV infection-driven airway inflammation on skeletal muscle dysfunction in the presence or absence of CS, by utilising a well-established preclinical model of AECOPD (Vlahos and Bozinovski, 2014) and a novel conditioned medium (CM) *in vitro* approach (Mohammadipoor et al., 2018).

## Materials and Methods

### Animals

Because of the strain dependence on the development of respiratory disease following cigarette smoke (CS) exposure (Vlahos et al., 2006), male Balb/C mice were chosen for this experiment. 7-week-old male BALB/c mice were obtained from the Animal Resources Centre Pty. Ltd. (Perth, WA, Australia) and allowed to acclimatize for 1 week at the RMIT Animal Facility (Bundoora, VIC, Australia). Mice were housed in micro-isolator cages with an ambient temperature of 21°C under a 12:12 light/dark cycle (lights on at 7a.m. and off at 7p.m. AEST). Water and standard mouse chow were available ad libitum. Body weight was monitored 3 times a week. All experimental procedures and protocols were conducted in accordance with the Australian Code of Practice for the Care and Use of Animals for Scientific Purposes, and approved by the RMIT University Animal Ethics Committee (AEC 1533).

### Influenza A Virus Amplification

Mem71 (H3N1) is a strain of IAV with intermediate virulence, which is a result of genetic reassortant of A/Memphis/1/71 (H3N2) × A/Bellamy/42 (H1N1) (Reading et al., 1997; Gualano et al., 2008). The virus was grown in Madin-Darby canine kidney (MDCK) cells, which were maintained at 37°C/5% CO_2_ in RPMI 1640 medium containing 2 mM L-glutamine, 2 mM sodium pyruvate, 30 μg/ml gentamycin, 100 I.U./ml penicillin, 100 μg/ml streptomycin and 10% v/v heat inactivated FCS (Thermo Fisher Scientific, Massachusetts, United States). For viral amplification, 90% confluent MDCK cells were washed twice in PBS and infected with egg grown virus at 0.01 plaque forming units/cell. After 1.5 h, the inoculum was removed, cells were washed twice with PBS, followed by the addition of a low protein, serum free medium (VP-SFM, Thermo Fisher Scientific, Massachusetts, United States) containing 0.5 μg/ml trypsin. Virus was harvested 48 h later, when there was extensive viral cytopathic effect. Flasks were shaken to loosen cells, the medium and cells were collected, briefly vortexed, subjected to a low-speed clearing spin and the clarified supernatant was collected and stored in aliquots at -80°C until required.

### Cigarette Smoke Exposure and Influenza A Virus Infection

Mice were placed in 18L Perspex containers in a standard fume hood and exposed to smoke generated from three cigarettes (Winfield Red, Phillip Morris, VIC, Australia; the average particulate matter was 419 mg/m^3^) over a 1-h period. CS was generated in 60 ml tidal volumes over a 10 s period in order to mimic the inhalation rates observed in human smokers. Mice were exposed to CS in this fashion 3 times a day (15 min per cigarette), with a 2-h break between smoke sessions (9 cigarettes per day total). CS exposure occurred for 5 days a week for 8 weeks. Control animals (Sham) were also placed in 18L Perspex containers but were not exposed to CS. At the end of 8 weeks of CS exposure, mice were anesthetized with methoxyflurane (Medical Developments International, VIC, Australia) and intranasally inoculated with either sterile phosphate buffered saline (PBS; diluent) or influenza A virus (Mem71/H3N1 strain at 1 × 10^4.5^ plaque forming units) dissolved in PBS. Following infection, CS exposure was ceased and mice were monitored periodically until the end of experiment for signs of distress (slowed respiration, reduced activity) and culled if symptoms persisted.

### Locomotor Activity Assessment

One day prior to termination, the locomotor activity of the mice was assessed in an open field arena. Locomotor activity was assessed between 8 a.m. and 1 p.m. to limit potential impacts of circadian rhythm. Mice were placed in the center of an empty 60 cm × 60 cm x 60 cm black plywood box and allowed to freely explore the environment for 8 min. The open field arena was cleaned with 70% ethanol prior to use and before each subsequent animal tested. The activity of the mice was filmed with a camcorder positioned above the arena (Panasonic HC-W585M, Osaka, Japan) and later scored on Ethovision XT software (v11.5; Noldus Information Technology, Wageningen, Netherlands). Briefly, a single zone was created in Ethovision that corresponded with the entire box. Mice were scored for total distance travelled within the zone during an 8-min period and trace maps were generated.

### Muscle Function Testing

At the end of the protocol, mice were anesthetized with a mixture of ketamine (80 mg/kg)/xylazine (16 mg/kg) and incisions were made to expose the tibialis anterior (TA) muscle, with care taken to avoid cutting the fascia as previously described (Chan et al., 2021). Briefly, the distal tendon was separated from the surrounding fascia in order to free the tendon from the ankle joint. The mouse was transferred to a heated platform (37°C) attached to an *in situ* contractile apparatus (1300A Aurora 3-in-1 whole animal system; Aurora Scientific, Ontario, Canada) and the limb of interest was secured to the platform via a foot clamp and a needle inserted through the patellar tendon and into the platform. The separated tendon was tied and secured to an isometric force transducer via suture threads. Two fine electrodes were inserted into the muscle belly ∼3–5 mm apart and penetrating just below the surface of the muscle. The muscle was periodically bathed with warm saline to prevent drying.

The TA was stimulated twice with a sustained contraction at 100 Hz with a 2-min rest interval in between, in order to settle the muscle and tighten the knots. Optimal muscle length (L_0_) was determined by increasing the length of the muscle with a micromanipulator. The muscle was stimulated with twitch contractions (single action potential allowing contraction then relaxation) every 30 s until a repeatable maximum peak twitch force was obtained. L_0_ was measured using precision digital calipers as the distance between the distal myotendinous junction and the insertion of the TA at the base of the knee. A force frequency test was performed which involved contractions induced at increasing frequencies (10, 20, 30, 50, 80, 100, 150, 200, 250, and 300 Hz) at 2-min intervals. Forces were converted to a digital signal and recorded by DYNAMIC MUSCLE ANALYSIS 611ATM (Aurora Scientific).

### Tissue Sample Collection

Mice were euthanized with sodium pentobarbitone (240 mg/kg; Virbac Australia, NSW, Australia) *via* intraperitoneal injection. Bronchoalveolar lavage fluid (BALF) was collected by flushing out the lungs with 400 µL ice-cold PBS followed by three aliquots of 300 µL ice-cold PBS, generating ∼1 ml of BALF per mouse. The total number of viable cells in the BALF was measured by diluting 50 µL of BALF with 50 µL of an acridine orange stain (Invitrogen, VIC, Australia). Cells were counted on a standard Neubauer hemocytometer under fluorescent light with an Olympus BX53 microscope (Olympus, Tokyo, Japan). To differentiate between the cell populations in the BALF, 50,000 cells were centrifuged using ∼50–200 µL of BALF at 400rpm for 10 min on a Shandon Cytospin 3 (Thermo Fisher Scientific, Massachusetts, United States). Dried cytospots were fixed with Shandon^TM^ Kwik-Diff^TM^ fixative (Thermo Fisher Scientific, Massachusetts, United States) and stained with Hemacolor Rapid Red and Blue dye (Merck, Australia). Cells were identified and differentiated into macrophages, lymphocytes, and neutrophils according to standard morphological criteria, with a minimum of 500 cells counted per slide. Hind limb muscles (quadriceps, TA, calf muscles) were dissected from each mouse, weighed, snap frozen in liquid nitrogen, and stored at −80°C until required. Muscles for morphological analysis were frozen in Tissue-Tek O.C.T compound (ProSciTech, QLD, Australia) under liquid nitrogen cooled isopentane, then stored at -80°C until required.

### Immunohistochemical Analyses

Frozen O.C.T embedded muscles were sectioned in a cryostat (Leica Biosystems, United States) for immunofluorescent staining of slow skeletal muscle myosin heavy chain (SM1; Santa Cruz Biotechnology Inc., TA, United States) and laminin (Novus Biologicals, Colorado, United States), as previously described (Chan et al., 2020) with slight modifications. Briefly, sections were fixed in 4% paraformaldehyde in 0.1 M phosphate buffer for 10 min at room temperature. Slides were then rinsed with 1X PBS +0.1% Tween® 20 (PBST) and incubated with blocking buffer (5% fetal bovine serum, 0.5% Triton-X, 0.01% sodium azide, 1X PBS) for 2 h at room temperature. Fluorophore conjugated antibodies for SM1 and laminin were added to sections and allowed to incubate overnight at 4°C. Myofiber cross-sectional area was determined by staining sections for laminin. Muscle fiber typing was determined by staining muscle sections for SM1. Following overnight incubation, sections were washed with 1X PBST and slides were mounted with Fluoromount-G™ Mounting Medium, with DAPI (Thermo Fisher Scientific, Massachusetts, United States) and cover slipped.

Slides were viewed and imaged using an Olympus Slide Scanner (VS120-S5, Olympus, United States) and analyzed using cellSens™ Life Science Imaging Software (Olympus, United States). Briefly, user defined boundaries consistent across each sample (size and location) were created and the cross-sectional area of fibers within boundaries was measured using the cellSens^TM^ measuring tool. Fiber types were identified based on the staining color and oxidative myofibers were manually counted and expressed as a percentage of all fibers within boundaries.

### Cell Culture and Conditioned Media Study

Human bronchial epithelial cell line, BEAS-2B (CRL-9609; American Type Culture Collection, United States), were grown as monolayers in a humidified incubator with 5% CO_2_ at 37°C in LHC-9 medium (Thermo Fisher Scientific, Massachusetts, United States) supplemented with 50 μg/ml gentamycin and 10% fetal calf serum (Thermo Fisher Scientific, Massachusetts, United States), with medium changes every 2–3 days. C2C12 murine myoblasts (CRL-1772, American Type Culture Collection) were cultured in high glucose Dulbecco’s Modified Eagle’s Medium (DMEM; Thermo Fisher Scientific, Massachusetts, United States) supplemented with 1% penicillin/streptomycin (100 units/mL penicillin and 100 μg/ml streptomycin; Thermo Fisher Scientific, Massachusetts, United States) and 10% fetal bovine serum (FBS; Thermo Fisher Scientific, Massachusetts, United States). Cells were cultured in a T-75 flask and were passaged at 70–80% confluence. The flasks were kept in a humidified incubator at 37°C with 5% CO_2_. To induce differentiation, confluent monolayers of C2C12 myoblasts were switched to differentiation medium (DM) consist of high-glucose DMEM supplemented with 1% penicillin/streptomycin and 2% horse serum (Thermo Fisher Scientific, United States) and the DM was changed daily. All experiments were performed on day 6 when majority of myoblasts have transformed into mature myotubes.

Cigarette smoke extract (CSE) was generated as previously described (Chan et al., 2021) with slight modifications. Briefly, one cigarette (Winfield Red, Phillip Morris International, Australia) was bubbled through a 25 ml of pre-warmed experimental media at a rate of 3 ml/s to produce 100% CSE stock solution. The stock solution was sterile filtered and diluted to obtain desirable concentration for experimentation. For the CM study, BEAS-2B cells were stimulated with either polyinosinic:polycytidylic acid (poly I:C, 4 μg ml^−1^), 25% CSE, or the combination of the two for 6 h. At the end of the stimulation, the media from the respective conditions were harvested and centrifuged at 400 g, 5 min at 4°C. The supernatants representing the CM were transferred onto differentiated C2C12 myotubes for 18 h.

### Quantitative Real-Time PCR

Total RNA was extracted from muscle tissue and cells using the RNeasy® Mini Kit (Qiagen, Germany) according to manufacturer instructions. RNA was reverse transcribed to cDNA using the High-Capacity RNA-to-cDNA Kit (Thermo Fisher Scientific, Massachusetts, United States) according to manufacturer instructions and real time PCR reactions were performed on the QuantStudio 7™ (Applied Biosciences, United States) using mouse specific TaqMan ® Gene Expression Assays: *Igf-eb* (AIKALFT)*, Mstn* (Mm01254559_m1)*, Cybb* (Mm01287743_m1)*, Gpx1* (Mm00656767_g1)*, Il-6* (Mm00443258_m1)*,* and *Tnfα* (Mm00446190_m1). All reactions were performed in triplicate and data obtained was normalized against glyceraldehyde 3-phosphate dehydrogenase (GAPDH), used as the reference gene, prior to analysis using the delta-delta Ct method.

### Western Blotting and Oxyblot

Tissue samples were homogenized in 500 µL of ice cold RIPA lysis buffer [150 mM NaCl, 1% Triton X-100, 0.5% Na-deoxycholate, 0.1% sodium dodecyl phosphate, 50 mM Tris (pH 8)] supplemented with β-mercaptoethanol (1%) and protease and phosphatase inhibitor cocktail (1%, Cell Signaling Technology, United States). The samples were incubated on ice for 30 min before being centrifuged for 10 min at 14,000 g at 4°C and supernatant was collected for immediate use or stored at −80°C. Protein concentrations were quantified through the use of a commercially available colorimetric bicinchoninic acid (BCA) protein assay kit (Thermo Fisher Scientific, Massachusetts, United States), following manufacturer instructions. For western blots, protein samples were denatured in SDS loading buffer (4x Laemmli buffer; 50 mM Tris-HCl pH 6.8, 2% SDS, 10% glycerol, 1% β-mercaptoethanol, 12.5 mM EDTA, 0.02% bromophenol blue). Western blot analyses were performed on 12% SDS PAGE gels to assess key signalling molecules including phospho-/pan- STAT1, phospho-/pan- JNK, GAPDH, BiP, phospho-/pan-eIF2α, phospho-/pan-p70S6 kinase, phospho-/pan- S6rp, phospho-/pan- 4E-BP1, tubulin (Cell Signaling Technology, United States), MAFbx and MURF-1 (Abcam, United States). For oxyblots, protein samples were derivatized and stabilized using the OxyBlot Protein Oxidation Detection kit (Merck, Massachusetts, United States) for immunoblot detection of carbonyl groups, following manufacturer instructions. Membranes were developed using chemiluminescent substrates and imaged using the ChemiDoc system (BioRad Laboratories Inc., United States). Quantitative densitometry analysis of bands of interest was performed using Image Lab software (Ver. 6.0, Bio-Rad Laboratories Inc.).

### Statistical Analyses

All data are presented as mean +SEM unless otherwise stated; *n* represents the number of mice. Multivariate analysis was performed *via* two-way ANOVA, followed by Tukey *post hoc* test where significant differences were found. All statistical analyses were performed using PRISM 9^TM^ (GraphPad Software, CA, United States). In all cases, statistical significance was assumed when *p* < 0.05.

## Results

### IAV Infection Exacerbates Airway Inflammation Without Causing Skeletal Muscle Loss

**FIGURE 1:**
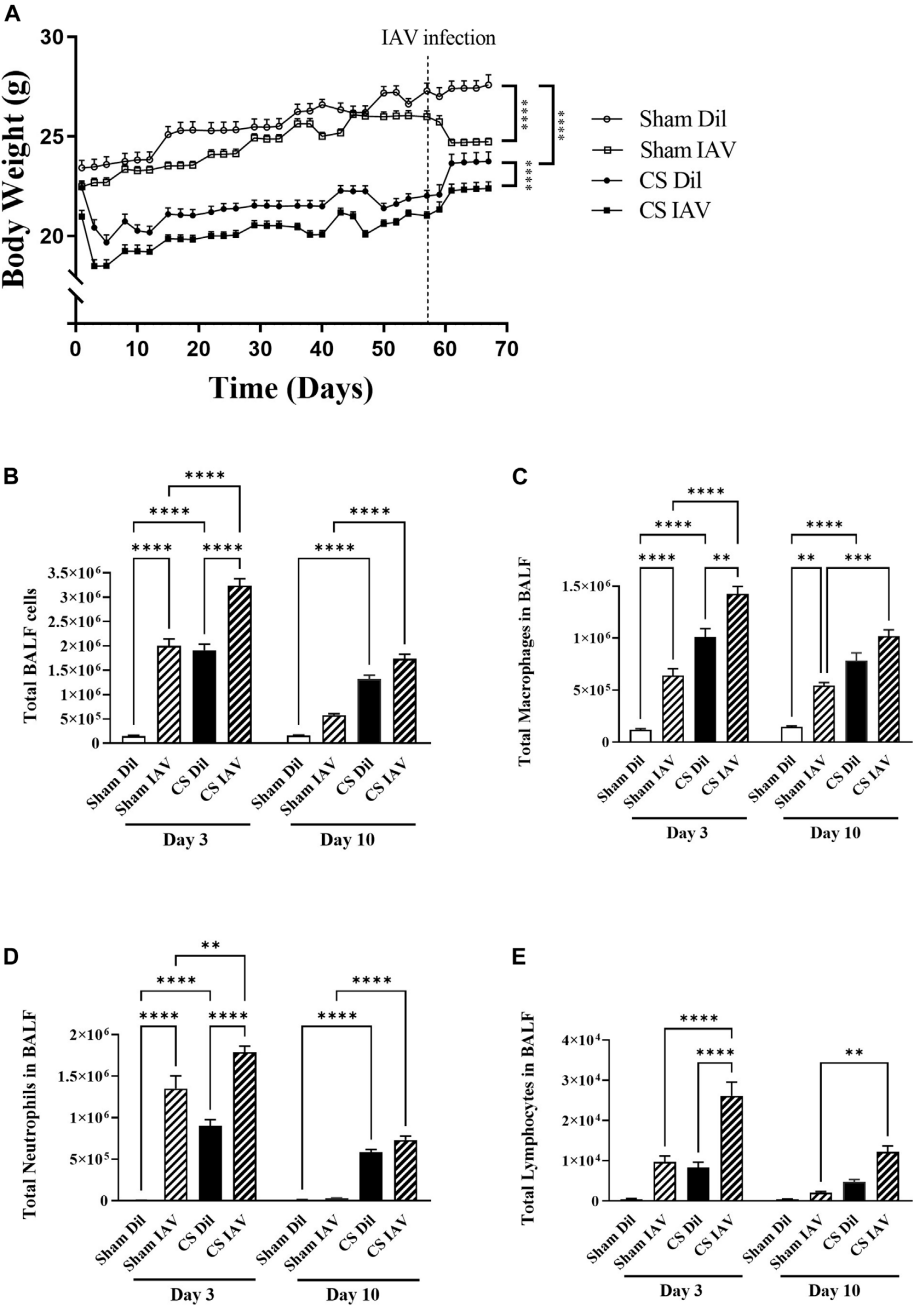
Effect of CS exposure and influenza A virus (IAV) infection on body weight and BALF cellularity. **(A)** Progressive body weight of mice across the experiment, and number of **(B)** total cells, **(C)** macrophages, **(D)** neutrophils and **(E)** lymphocytes in the bronchoalveolar lavage fluid in CS-exposed and Sham mice following IAV infection. Data shown as mean +SEM (*n* = 7 per group). **p < 0.01, ***p < 0.001, ****p < 0.0001.

CS exposure suppressed body weight gain (∼2 g lower than Sham) which was maintained throughout the course of the experiment (Figure 1A). Following IAV infection, Sham mice experienced a ∼9% reduction in body weight compared to the non-infected Sham mice. Under CS condition, IAV infection also caused a similar reduction in body weight. CS exposure or IAV alone produced a robust increase in total cellularity within the BALF (Figure 1B). This was attributed to an increase in macrophage, neutrophil, and lymphocyte counts (at day 3). This was further augmented by the combination of CS exposure and IAV infection (Figures 1B–E) recapitulating that of AECOPD in human patients (Bozinovski et al., 2012; Wang et al., 2019). Similar to our previous observations (Oostwoud et al., 2016), the IAV infection-driven exacerbation was characterized by a peak in airway inflammation at day 3 that was resolved by day 10, marking the resolution phase of infection.

**FIGURE 2:**
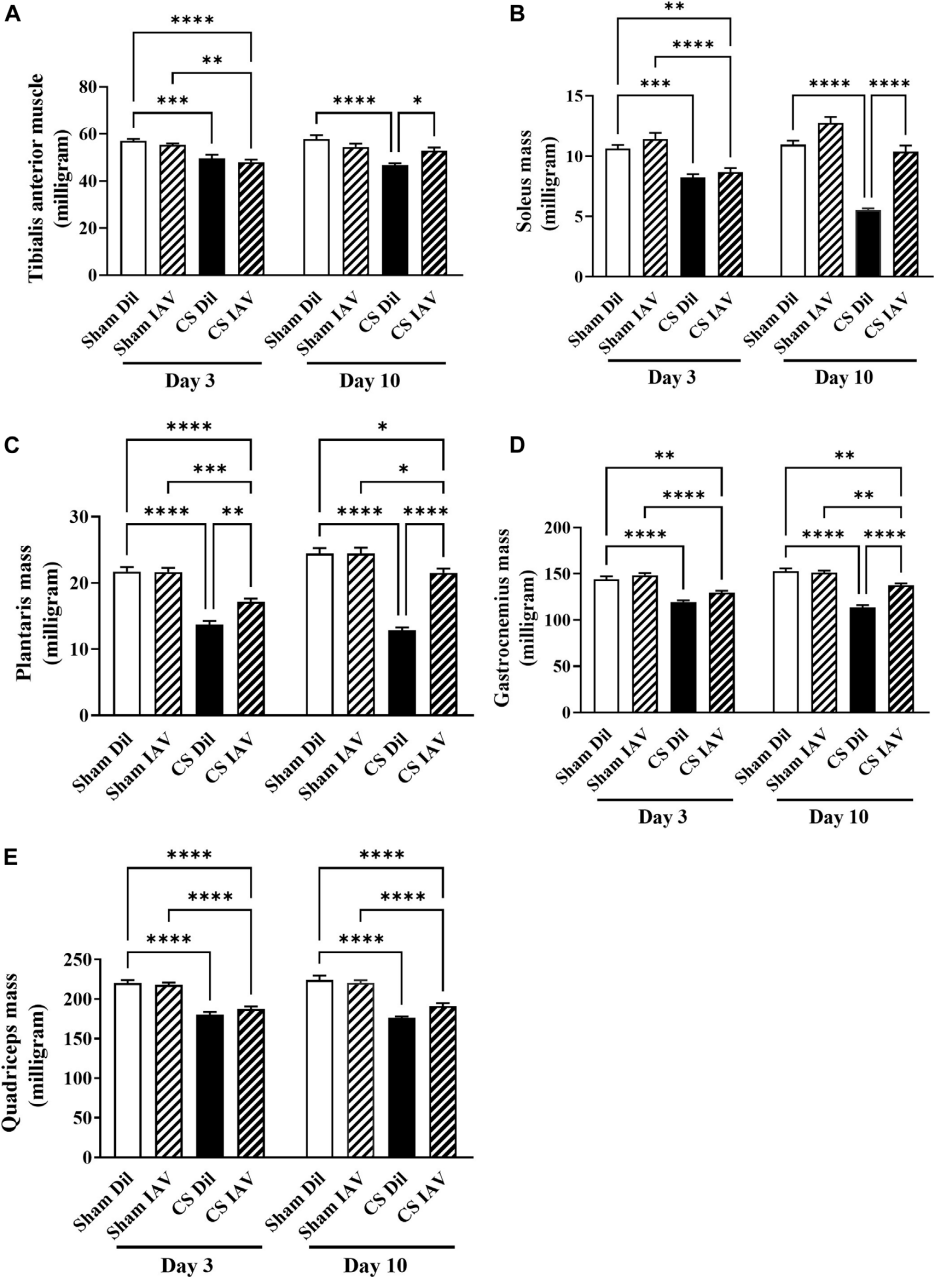
Effect of CS exposure and influenza A virus (IAV) infection on mass of hind limb muscles. The weight of the **(A)** tibialis anterior, **(B)** soleus, **(C)** plantaris, **(D)** gastrocnemius, and **(E)** quadriceps muscles from CS-exposed and Sham mice following IAV infection. Data shown as mean +SEM (*n* = 14 per group). *p < 0.05, **p < 0.01, ***p < 0.001, ****p < 0.0001.

CS exposure caused a global reduction in mass of the hind limb muscles (Figures 2A–E), which was sustained throughout the course of the experiment. This was characterized by a 13 and 30% decrease in mass of the TA (Figure 2A) and plantaris (Figure 2C) respectively, which are predominated by fast-twitch fibers. Similar trends were observed in the slow-twitch fiber predominant soleus with a 22% decrease observed (Figure 2B), along with a 17% loss in both the mixed-fiber predominant gastrocnemius and quadriceps (Figures 2D,E). Despite the exacerbated airway inflammation, the combination of CS and IAV infection (at day 3) did not result in any further loss of muscle mass. By day 10, most of the muscle loss driven by the combination of CS exposure and IAV infection returned to Sham level (Figures 2A–E), suggesting viral exacerbation is unlikely to have additive effects on CS-induced muscle loss.

### IAV Infection Decreased Myofiber Area Irrespective of CS Exposure

**FIGURE 3:**
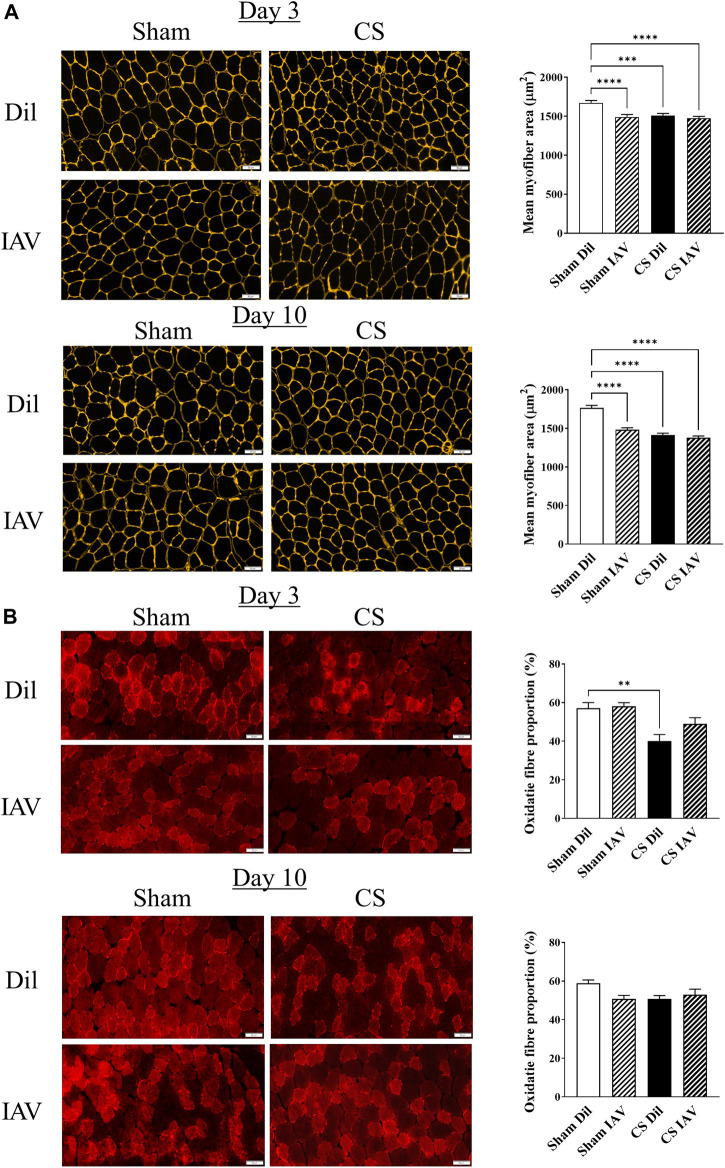
Effect of CS exposure and influenza A virus (IAV) infection on morphology of tibialis anterior (TA) muscle. Immunofluorescent examination and quantitative data of **(A)** myofiber cross-sectional area with laminin staining and **(B)** fiber distribution with slow myosin heavy chain staining, in cross-sectioned TA of CS-exposed and/or IAV infected mice 3- and 10-days post infection. Scales bars: 50 µm. At least 600 fibers were counted per animal. Data shown as mean +SEM (*n* = 5 per group). **p < 0.01, ***p < 0.001, ****p < 0.0001.

In line with the loss of TA muscle mass, CS exposure also caused a ∼10% reduction in mean myofiber cross−sectional area (CSA), which persisted throughout the course of experiment (Figure 3A). Despite the seemingly unaffected muscle mass, IAV infection also resulted in a similar reduction in mean myofiber CSA, in the Sham group. However, the suppressive effects of IAV infection on mean myofiber CSA did not appear to be alleviated on day 10, unlike that of muscle mass (Figure 3A). Furthermore, immunostaining detected a significant reduction in the proportion of oxidative muscle fibers following CS exposure, suggesting the presence of a slow-to-fast fiber type shift, like that seen in people with stable COPD (Gosker et al., 2007). In contrast, IAV infection did not significantly impact fiber type composition, irrespective of CS exposure or time after infection (Figure 3B), suggesting viral exacerbation is unlikely to contribute to fiber type transformation of limb muscle.

### IAV Infection Exacerbated CS-Induced Contractile Impairment Independent of Locomotor Activity

**FIGURE 4:**
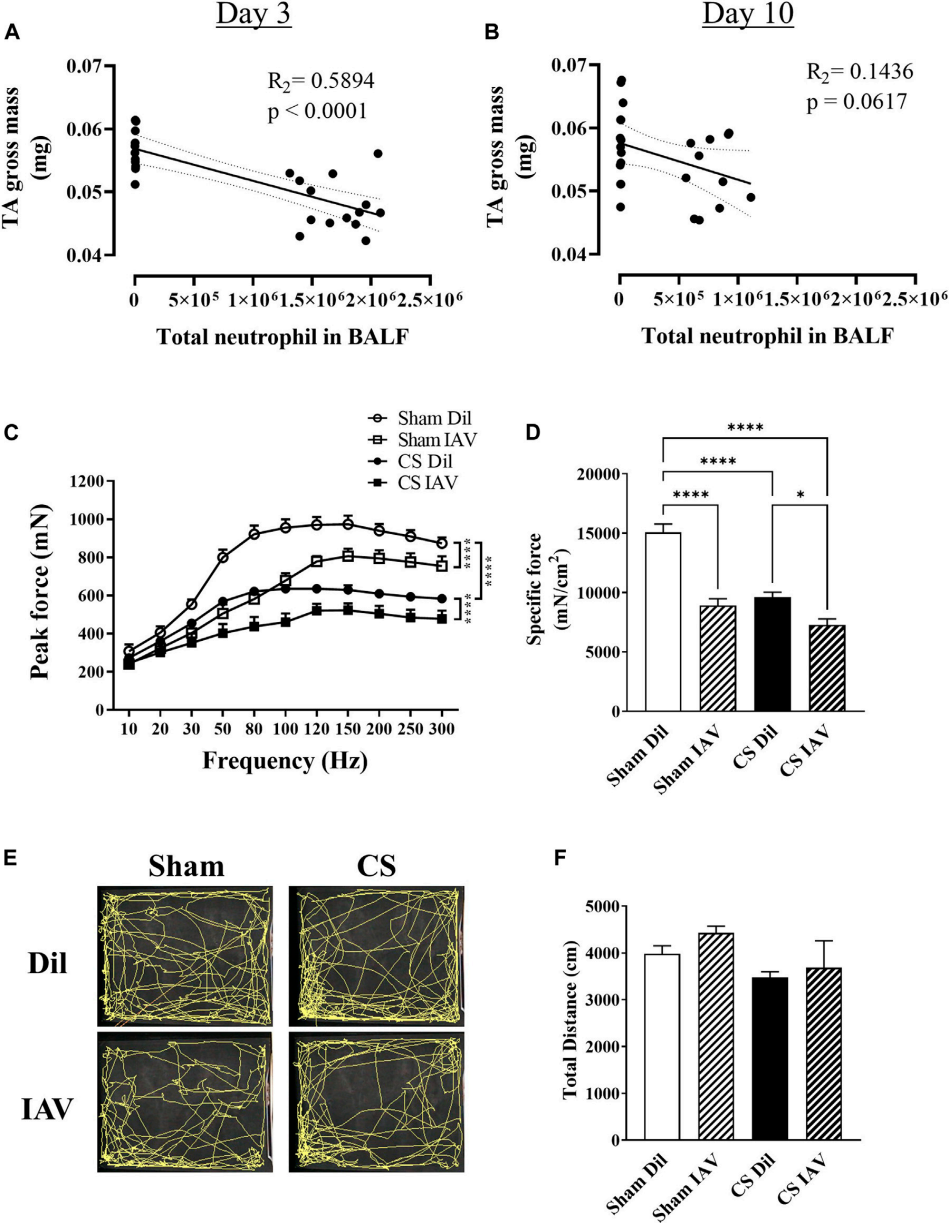
Effect of CS exposure and influenza A virus (IAV) infection on tibialis anterior (TA) muscle function. Correlational analyses of TA gross weight and total neutrophil counts in the BALF at **(A)** day 3 and **(B)** day 10 post IAV infection in CS-exposed and Sham mice. **(C)** Maximum contractile force and **(D)** specific force at 120 Hz were determined at peak of airway inflammation i.e., day 3. Prior to muscle functional analyses, recordings were made of locomotor activity over an 8-min period and **(E)** trace maps were generated and analyzed for total distance travelled (cm). Data shown as mean +SEM (*n* = 6 per group for correlational and TA contractile function analyses, and n = 10 per group for locomotor activity assessment). *p < 0.05, ****p < 0.0001.

To investigate the impacts of viral exacerbation on limb muscle function, we performed detailed functional analysis on day 3, when airway inflammation was at its peak (Figures 1B–E). Our correlational analyses revealed that the loss of TA mass is directly correlated with the peak increase (i.e., day 3) in BALF neutrophilia (Figure 4A), which is a common characteristic of both stable COPD and AECOPD (Gompertz et al., 2001; Hoenderdos and Condliffe, 2013). Although a similar correlative trend was observed on day 10, the R_2_ value was much lower (Figure 4B), suggesting the main effects of viral exacerbation on limb muscle are most likely to occur on day 3. Our muscle function analysis found that either IAV infection or CS exposure caused a significant reduction in contractile function of the TA muscle, which was further deteriorated when IAV infection was combined with CS exposure (Figure 4C). In line with this, the specific force analysis demonstrated a 41 and 36% decrease in contractile function by IAV infection and CS exposure respectively when compared to Sham animals, while a 52% decrease was observed when IAV infection was combined with CS exposure (Figure 4D), suggesting the manifestation of muscle weakness. As muscle weakness is closely linked to reduced physical activity levels (Degens and Alway, 2006; Passey et al., 2016), we assessed the locomotor activity of the mice using an open field arena. Despite its prominent effect on muscle strength, IAV infection had no apparent effect on total distance travelled, irrespective of CS exposure (Figure 4E), hence physical inactivity is unlikely to contribute to the observed muscle weakness. These findings suggest that viral exacerbation primarily impacts muscle strength rather than mass, and this is not compounded by changes in locomotor activity.

### IAV Infection Evoked Oxidative Stress but did not Potentiate CS-Induced Disruption in Myogenic Signaling

**FIGURE 5:**
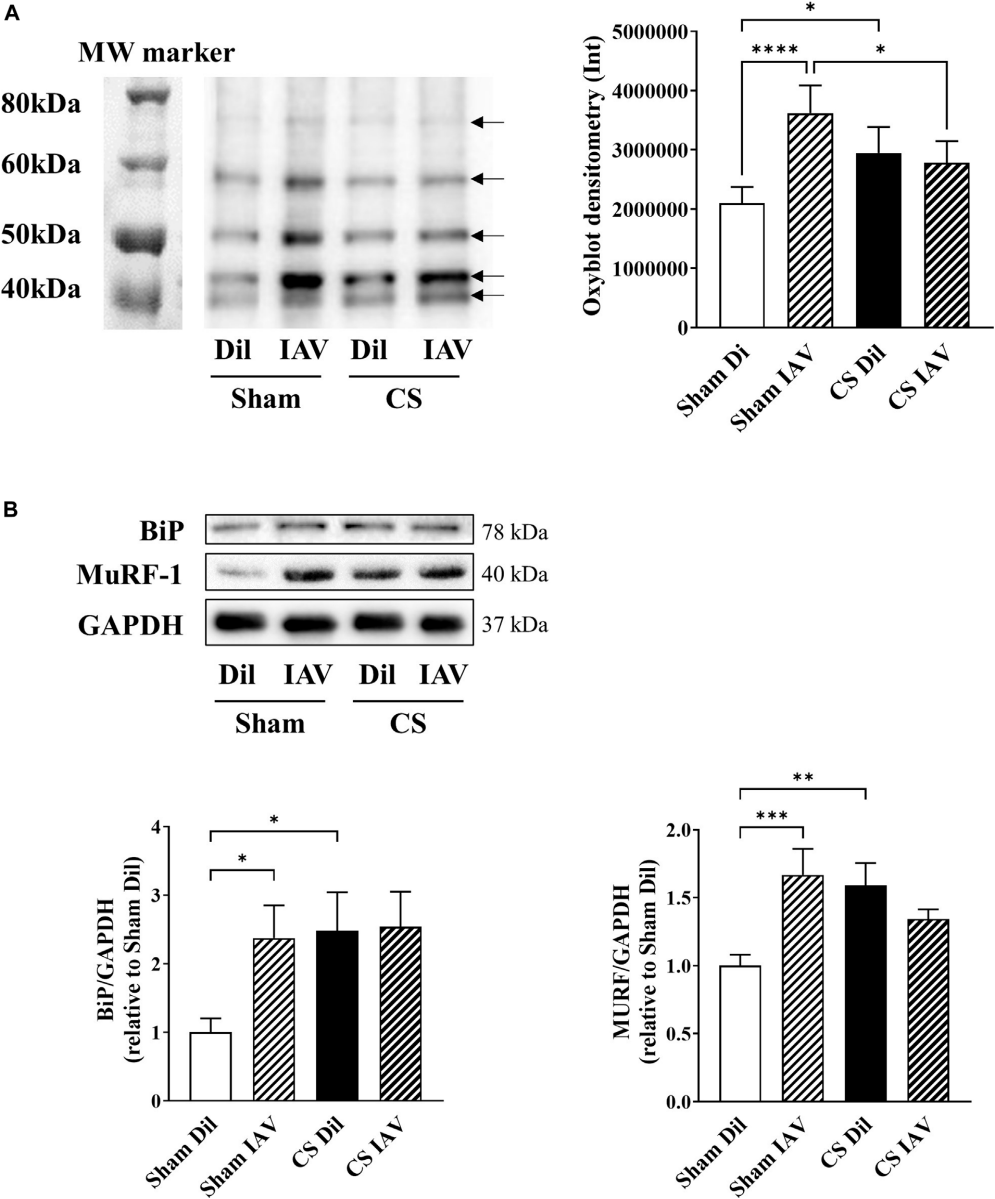
Effect of CS exposure and influenza A virus (IAV) infection on oxidative stress and myogenic signaling in tibialis anterior (TA) of mice. Total protein carbonylation was measured using **(A)** Oxyblot staining and subsequent densitometry analysis on muscle homogenates of CS-exposed and Sham mice infected with IAV. Bands indicated by black arrows in densitometry were measured and added up for analysis. **(B)** Western blot images and quantitative densitometry analysis of BiP and MURF. Data shown as mean +SEM (*n* = 8 per group). *p < 0.05, **p < 0.01, ***p < 0.001, ****p < 0.0001.

Previous studies from our laboratory (Chan et al., 2021) and others (Barreiro et al., 2010; Fermoselle et al., 2012) have demonstrated the involvement of oxidative stress in CS-induced muscle dysfunction, however, its role in IAV infection remains unclear. An oxyblot assay on the TA muscle detected a 60% increase in protein carbonylation following IAV infection in Sham mice, and this was not further elevated when combined with CS exposure (Figure 5A). At the molecular level, IAV infection elicited a 15-fold increase in *Cybb* [NADPH oxidase 2 (Nox2)] and a 3-fold increase in glutathione peroxidase 1 (*Gpx-1)* mRNA expression. However, this response appeared to be completely blunted when IAV infection was combined with CS exposure (Table 1). IAV infection also subdued myogenic signaling, characterized by a downregulation in mRNA expression of myogenin (*Myog;* 60% reduction), a positive regulator of myogenesis, and concomitant upregulation of myostatin (*Mstn*; 10-fold), which inhibits myogenesis, irrespective of CS exposure (Table 1). In line with the induction of oxidative stress and blunted myogenic signaling, the protein abundance of binding immunoglobulin protein (BiP) and muscle RING-finger protein-1 (MuRF-1) were markedly elevated by IAV infection, irrespective of CS exposure (Figure 5B). BiP is a cellular redox sensor and a master regulator of protein folding (Wang et al., 2014), while MuRF-1 is a downstream effector of myostatin that promotes ubiquitin-mediated protein degradation in skeletal muscle (Desgeorges et al., 2017). These findings imply that IAV infection does not further exacerbate the oxidative burden in the muscle or increase the disruption to myogenic signaling incurred by CS exposure.

**TABLE 1:** Summary of gene expression data from the TA muscle (mean ± SEM).

Gene name	Sham Dil	Sham IAV	CS Dil	CS IAV
*Cybb*	1.02 ± 0.05	15.39 ± 1.71^†††^	1.08 ± 0.06	2.46 ± 0.24
*Gpx−1*	1.11 ± 0.12	2.92 ± 0.31^†††^	1.90 ± 0.43	1.03 ± 0.11^***^
*Myog*	1.15 ± 0.19	0.41 ± 0.07^†††^	0.73 ± 0.04^*^	0.33 ± 0.07^† §^
*Mstn*	1.08 ± 0.10	10.69 ± 1.57^†††^	17.15 ± 1.37^***^	5.73 ± 0.93^*^^††† §§^
*Myh7*	1.07 ± 0.11	0.65 ± 0.076^††^	0.18 ± 0.02^***^	0.40 ± 0.08^*^^§§§^
*Tpm3*	1.11 ± 0.10	2.13 ± 0.34^†^	2.15 ± 0.17^*^	2.27 ± 0.28^§^

* *p* < 0.05, *** *p* < 0.001 vs. the respective Sham; ^†^ *p* < 0.05, ^††^ *p* < 0.01, ^†††^ *p* < 0.001 vs. the respective diluent−treated Sham/CS; ^§^ *p* < 0.05, ^§§^ *p* < 0.01, ^§§§^ *p* < 0.001 vs. Sham Dil.

### Exposure of Bronchial Epithelial Cells to TLR3 Activating Viral Mimetics Elicits STAT1 Activation and Accentuated the Expression of Pro-Inflammatory Cytokines

**FIGURE 6:**
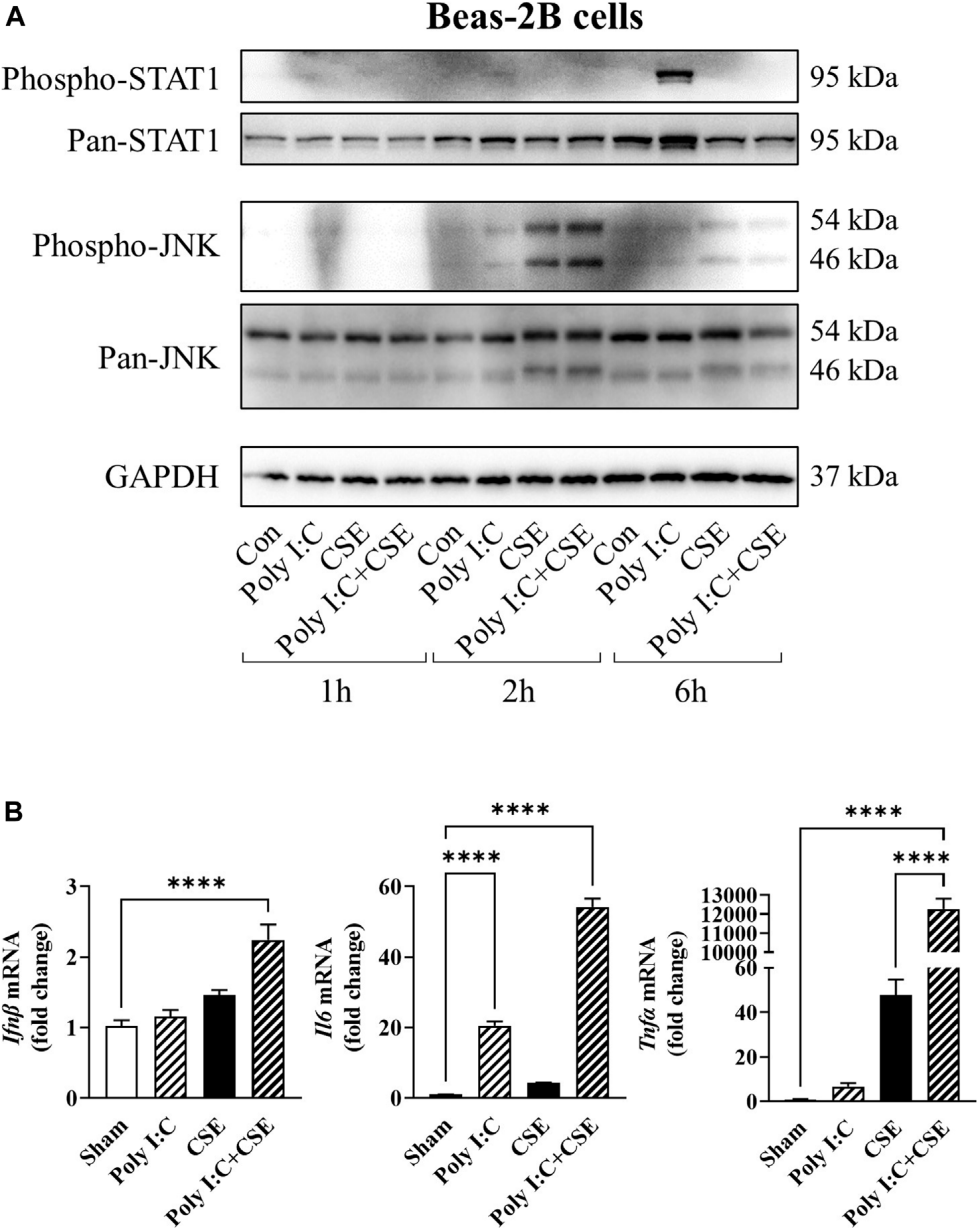
Effect of CSE and poly I:C on inflammatory response of lung epithelial cells. BEAS2B cells were exposed to polyinosinic:polycytidylic acid (poly I:C), 25% cigarette smoke extract (CSE), or a combination of the two for 1, 2 and 6 h. **(A)** Western blot images of phospo-STAT1, pan-STAT1, phospho-JNK and pan-JNK. **(B)** qPCR analysis was performed to assess expression of inflammatory markers *Ifnβ, Il6* and *Tnfα.* Data shown as mean +SEM (*n* = 7–9 per group). ****p < 0.0001.

To ascertain the contributory role of airway inflammation in the disruption of myogenic signaling, we treated bronchial epithelial cells (BEAS−2B) with poly I:C, a well-established viral mimetic used to exacerbate inflammation in lung injury models (Vella et al., 2020), in the presence or absence of CSE, to recapitulate a viral exacerbation. Poly I:C stimulated the phosphorylation of signal transducer and activator of transcription 1 (STAT1; Figure 6A) and resulted in a 20-fold increase in the expression of interleukin-6 (*Il-6*; Figure 6B). In contrast, CSE elicited rapid phosphorylation of c-Jun N-terminal kinase (JNK) which was accompanied by an increased expression of tumor necrosis factor α (*Tnfα*: Figures 6A,B). Although cell viability was seemingly unaltered (Supplementary Figure S1A), co-exposure to poly I:C and CSE resulted in a marked increase in interferon β (*Ifnβ*; 2.4-fold), *Il-6* (54-fold) and *Tnfα* (∼12,000-fold) mRNA expression, suggesting an amplified toll-like receptor (TLR)-3-driven inflammatory response in these bronchial epithelial cells.

### Amplified Lung Cell Inflammation did not Potentiate Myogenic Disruption Driven by CSE

**FIGURE 7:**
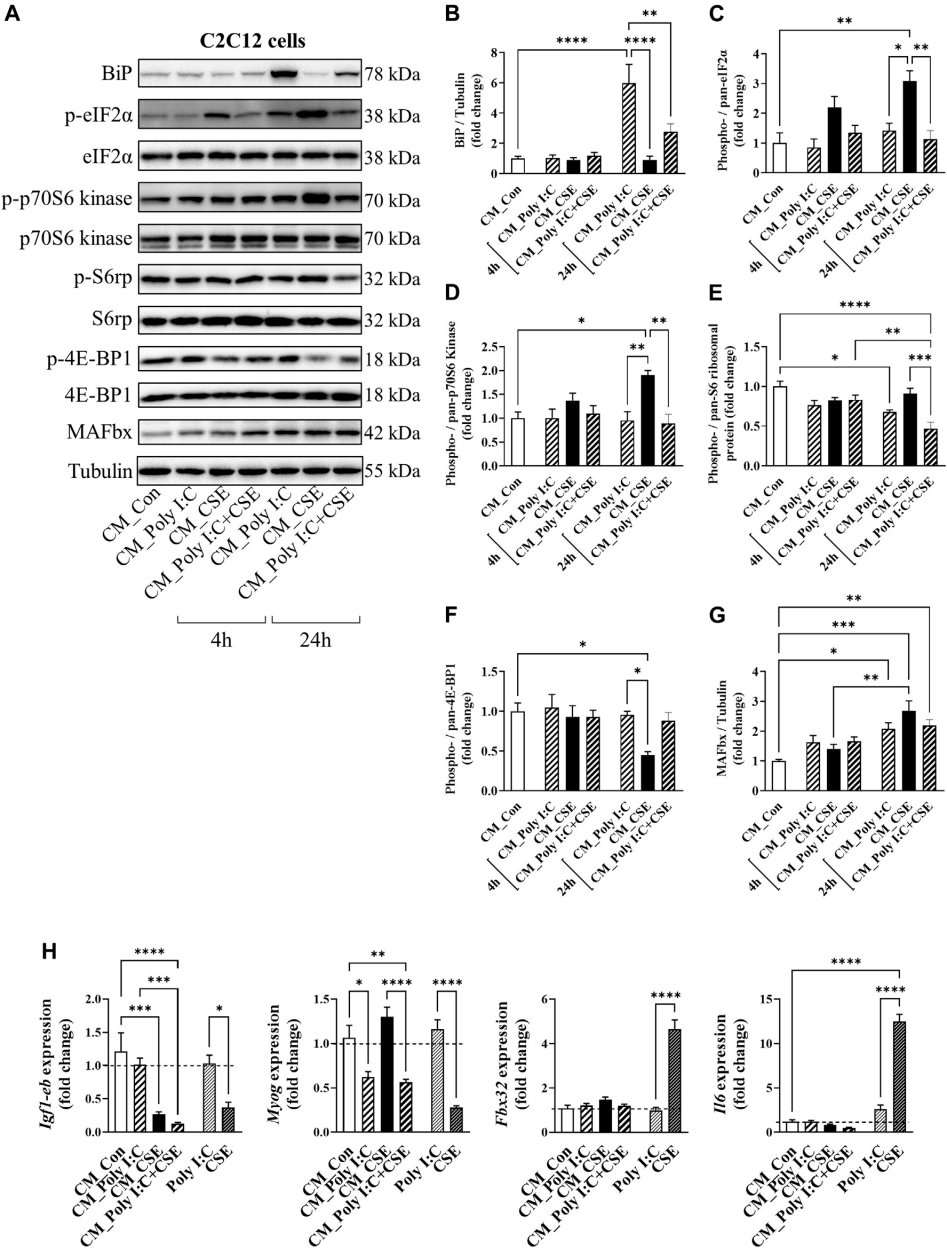
Effect of cigarette smoke extract (CSE) and poly I:C conditioned media (CM) on proteostatic signaling of C2C12 myotubes. CM derived from BEAS2B cells exposed for 6 h to polyI:C, 25% CSE or a combination of the two was transferred onto differentiated C2C12 myotubes. **(A)** Western blot images and densitometry analysis of **(B)** BiP, **(C)** phospho (ser51)-/pan-eIF2α, **(D)** phospho (Thr389)-/pan-p70S6 kinase, **(E)** phospho (Ser235/236)-/pan- S6 ribosomal protein, **(F)** phospho (Thr37/46)-/pan- 4E-BP1, and **(G)** MAFbx. **(H)** qPCR analysis was performed to assess expression of *Igf-1eb, Myog, Fbx32 and Il6.* Data shown as mean +SEM (*n* = 7–9 per group). *p < 0.05, **p < 0.01, ***p < 0.001, ****p < 0.0001.

To determine whether the TLR3-driven inflammatory response of the lung cells may potentiate myogenic disruption by CSE, we transferred the culture medium of the BEAS−2B cells (i.e., CM) onto C2C12 myotubes for a time-course experiment, as outlined in Figure 7. Myotubes treated acutely (4-h exposure), with CM from poly I:C-treated cells displayed no observable changes in key signal transducers of protein folding [BiP, eukaryotic initiation factor 2α (eIF2α)], protein synthesis [p70 S6 Kinase, S6 ribosomal protein, eukaryotic translation initiation factor 4E-binding protein 1 (4E-BP1)] or in muscle-specific F-box (MAFbx); a negative regulator of muscle mass (Passey et al., 2016). Despite not having an apparent effect on the structural integrity of the myotubes (Supplementary Figure S1B), 24-h exposure to the poly I:C CM resulted in a marked increase in BiP and eIF2α phosphorylation (Figures 7A–C), suppression of S6 ribosomal protein phosphorylation (Figures 7A,E), and elevation of MAFbx abundance (Figures 7A,G), suggesting a shift in myogenic balance. CM from CSE-exposed cells had distinct impacts on the myogenic signaling, characterized by an even greater phosphorylation of eIF2α without an increase in BiP protein expression (Figure 7B), increased p70 S6 Kinase phosphorylation (Figure 7D), diminished 4E-BP1 phosphorylation (Figure 7F) and a concomitant increase in MAFbx protein expression (Figure 7G). CM from the poly I:C + CSE-exposed lung cells increased BiP and MAFbx abundance, like the poly I:C alone experiments, and furthermore, significantly suppressed the phosphorylation of S6 ribosomal protein.

We next performed qPCR on the CM-exposed myotubes for markers of myogenesis and inflammation. Unlike CSE, direct stimulation of C2C12 myotubes with poly I:C had no significant impact on myogenic genes and *Il6* expression (Figure 7H). Similar to what was seen in our *in vivo* IAV infection model (Table 1), poly I:C CM caused a ∼50% reduction in *Myog* mRNA expression, without any significant changes observed in the gene expression of either *Fbx32*, which encodes for the MAFbx protein, or *Il6* (Figure 7H). Importantly, CM from poly I:C + CSE did not further increase these changes, suggesting the TLR3-driven inflammatory response of the lung cells is unlikely to have an addictive effect on myogenic disruption driven by CSE.

## Discussion

Skeletal muscle dysfunction is a common comorbidity amongst people with COPD and is often worsened during/following an acute exacerbation (Güerri et al., 2010; Greening et al., 2015; Perrot et al., 2020). The present study aimed to examine how acute exacerbation by IAV infection promotes muscle dysfunction, focusing on the contributory role of airway inflammation in this process. Our results demonstrated that IAV infection amplified CS-induced airway inflammation and limb muscle weakness without further impacting muscle mass. Although, IAV infection alone evoked oxidative stress in the TA, viral exacerbation did not further elevate oxidative stress levels or disruptions to myogenic signaling induced by CS exposure, suggesting airway inflammation is independent to the observed muscle loss. We next used an *in vitro* CM approach to further investigate this relationship. Despite an accentuated inflammatory response in the lung cells due to the activation of TLR3/STAT1, the combination of viral mimetics and CSE did not exert additive effects on myogenic signaling. Hence, airway inflammation is unlikely to be the sole driving force for the further deteriorated muscle dysfunction observed during AECOPD.

In COPD, neutrophilic recruitment into the lungs is directly correlated with disease severity and worsened respiratory symptoms including increased sputum production and breathlessness (Meijer et al., 2013). During infectious AECOPD, airway neutrophilia becomes even more prominent compared to stable COPD (Papi et al., 2006; Oostwoud et al., 2016), with recruited neutrophils being more activated, potentially driving further inflammation, and thereby increasing tissue damage and airway dysfunction (Johansson and Kirsebom, 2021). In line with this, our data showed that upon viral exacerbation, neutrophilia and total cellularity in the BALF were significantly increased at both day 3 and 10 timepoints, confirming the presence of an exuberant airway inflammation in our *in vivo* model that is characteristic of human AECOPD (Bozinovski et al., 2012; Wang et al., 2019).

Amplified airway inflammation evoked by infectious exacerbations have been shown to further the limb muscle loss seen in AECOPD (Perera et al., 2007; Rubinsztajn et al., 2014). In accordance with this, we detected a significant negative correlation between the loss of TA muscle mass and BALF neutrophilia on day 3, but not day 10 post-infection. This finding suggests there is a link between the peak of airway inflammation and the manifestation of muscle loss during AECOPD. However, the gross weight of the leg muscles was not further reduced following viral exacerbation, and this observation was substantiated by the lack of change in mean myofiber area, and the absence of further disruptions to myogenic signaling. This suggests a single episode of viral exacerbation may not necessarily add to the COPD-related muscle loss, despite the presence of an accentuated airway inflammation. Guerri *et al.* (Güerri et al., 2010) observed a greater loss of limb muscle CSA in individuals who suffered from four or more episodes of AECOPD compared to individuals who only experienced a single exacerbation, highlighting the compounding effect of repeated exacerbation on limb muscle impairment.

Our *in vitro* experiments revealed that viral exacerbation CM did not incur further myogenic disruption or impact on cell viability. Of note, myotube loss and degradation of sarcomere structures were found following 48 h of exposure to CSE CM, which was not observed following exposure to Poly I:C CM. Direct exposure to poly I:C resulted in no myogenic disruption, suggesting the muscle cells are unresponsive to poly I:C even though TLR3 has been reported to be expressed in myotubes (Cho et al., 2017). Taken together, these data support the notion that TLR3/STAT1-driven airway inflammation does not potentiate COPD-related muscle loss.

Another main finding of the present study is that viral exacerbation drove a further impairment in contractile strength of the TA muscle of CS-exposed mice, despite no further reduction in muscle mass or mean myofiber CSA. This effect was persistent after adjusting for differences in muscle size, thus confirming the additive impact of viral exacerbation on muscle weakness. The TA is the strongest dorsiflexor muscle of the foot and is critical to gait as dorsiflexion clears the foot off of the ground during the swing phase (Hardin and Devendra, 2022). Therefore, we assessed locomotor activity to ascertain whether this exacerbation-related muscle weakness impacted gait, which is believed to play a major role in the exercise limitation observed clinically in individuals with COPD (Maltais et al., 2014). Surprisingly, neither CS exposure nor viral exacerbation had a significant impact on locomotor activity, despite a >50% reduction in the force generating capacity of the TA.

This brings about several important observations: i) impairment in contractile capacity and muscle mass loss may precede the manifestation of changes in locomotor activity; ii) contractile function may be more sensitive to exacerbation-related impairments than muscle mass; iii) limb muscle dysfunction can occur independent of physical inactivity, which is conceptually important as physical inactivity is a major contributor to muscle dysfunction, and infectious exacerbations are known to make patients become even less active (Pitta et al., 2005). If exacerbation-driven airway inflammation itself does not directly worsen limb muscle dysfunction, the question remains as to what causes the further muscle impairments observed during AECOPD. The cause of muscle dysfunction in this context is likely to be multifactorial, involving both conventional and unconventional factors such as unintended weight loss, length of hospitalization, glucocorticoids use and epigenetics (Passey et al., 2016). However, one should also be mindful of the locomotion/postural differences between rodent (quadrupedal) versus human (bipedal), which implies that the locomotor impact of limb muscle weakness may be more significant in human than in mice.

Despite the multifactorial nature, muscle impairments in stable COPD and AECOPD can be effectively targeted with nutritional interventions and exercise therapy (Passey et al., 2016). However, both nutritional interventions and exercise therapy may not always be feasible in patients with severe respiratory or limb muscle impairments, thus making pharmacologic approaches an important modality of treatment (van Bakel et al., 2021). The use of anabolic hormones, such as testosterone has been demonstrated to acutely improve muscle loss and dysfunction in patients with COPD (Casaburi et al., 2004), presumably *via* the IGF-1 pathway (Passey et al., 2016). It should be noted that hormone replacement therapies are often associated with adverse side-effects and the long-term benefits of anabolic hormone supplement remain to be established. The observation that IGF-I is only positively related to muscle strength when circulating levels of IL-6 is low (Barbieri et al., 2003) raises an important notion that the effectiveness of anabolic hormone therapy may be negative influenced by systemic inflammation, which is a hallmark feature of advanced COPD(Hillas et al., 2016). Inhibition of protein degradation in muscle has been explored as a potential therapy, though inhibition of autophagy (Masiero et al., 2009) or ubiquitin-proteasome system (Kitajima et al., 2014) have paradoxically resulted in muscle loss. The strong involvement of oxidative stress in the pathophysiology of both lung and limb muscle dysfunction has prompted the importance of targeting oxidative stress. Indeed, recent data from our laboratory demonstrated that apocynin, a free radical scavenger and a Nox2 inhibitor, attenuated airway inflammation and muscle impairments by CS exposure (Chan et al., 2021), suggesting the effectiveness and feasibility of targeting oxidative stress by pharmacological means to treat lung and systemic manifestations of COPD.

In summary, the present study demonstrated that viral exacerbation may deteriorate muscle weakness without additive effects on muscle loss. This may be explained in part by the minimal impacts of TLR3-driven airway inflammation on muscle cells. Our data also showed that muscle cells are relatively unresponsive to direct TLR3 stimulation, thus ruling out the possibility of endogenous TLR3 signaling in driving myogenic disruption. The findings of the present study prompt further research questions, particularly surrounding 1) the differences in IAV strains, and 2) the compounding effect of repeated viral exacerbations. It is known that different strains of IAV have different pathogenicity, and the strain used in the present study (Mem71) is considered to be of intermediate pathogenicity compared to other strains (Ong et al., 2017). Of note, severe IAV pneumonia caused by the A/WSN/33 strain has been demonstrated to directly promote muscle loss in mice, in an IL-6 dependent manner (Radigan et al., 2019). Meanwhile repeated episodes of exacerbations are known to aggravate not only respiratory symptoms, but also systemic comorbidities such as muscle dysfunction (Anzueto, 2010). Of interest, even a single episode of IAV-induced exacerbation by the Mem71 strain has been shown to provoke airway hypersensitivity to future allergen challenge, despite the initial IAV-induced bronchoalveolar inflammation had long subsided (Looi et al., 2021), suggesting an increased risk for future exacerbations. Therefore, future studies should examine the strain discrepancies of IAV (including impacts of viral loading and their respective impacts to disease outcomes) and the compounding impacts of repeated exacerbations to provide a more complete picture of the impacts of AECOPD on limb muscle dysfunction.

## Data Availability

The original contributions presented in the study are included in the article/Supplementary Material, further inquiries can be directed to the corresponding author.

## Glossary

4E-BP1: eukaryotic translation initiation factor *4E*-binding protein 1
AEC: animal ethics committee
AECOPD: acute exacerbation of chronic obstructive pulmonary disease
ANOVA: analysis of variance
BALF: bronchoalveolar lavage fluid
BiP: binding immunoglobulin protein
CM: conditioned medium
COPD: chronic obstructive pulmonary disease
CS: cigarette smoke
CSA: cross-sectional area
CSE: cigarette smoke extract
eIF2α: eukaryotic initiation factor 2α
GM-CSF: granulocyte-macrophage colony-stimulating factor
Gpx1: glutathione peroxidase 1
IAV: influenza A virus
IFN-β (*Ifnb*): interferon β
IFN-γ: interferon γ
IL-6: interleukin 6
IL-8: interleukin 8
JNK: c-Jun N-terminal kinase
MAFbx: muscle-specific F-box
Mstn: myostatin
MuRF-1: muscle RING-finger protein 1
Myh7: myosin heavy chain 7
Myog: myogenin
Nox2: NADPH oxidase 2
O.C.T: optimum cutting temperature
PBS: phosphate-buffered saline
PFU: plaque forming units
PolyI:C: polyinosinic:polycytidylic acid
STAT1: signal transducer and activator of transcription 1
TA: tibialis anterior
TLR3: toll-like receptor 3
TNF-α: tumour Necrosis Factor α
Tpm3: tropomyosin 3
