# Supplementary material for: Influenza A Virus-Driven Airway Inflammation may be Dissociated From Limb Muscle Atrophy in Cigarette Smoke-Exposed Mice
Source: Front Pharmacol. 2022 Mar 18;13:859146. doi: 10.3389/fphar.2022.859146 (PMC8971713; doi:10.3389/fphar.2022.859146)
Supplement: Supplementary file 1 [file DataSheet1.PDF]

Influenza A virus-driven airway inflammation may be dissociated from limb muscle atrophy in cigarette smoke-exposed mice

Kevin Mou[1], Stanley MH. Chan[1], Kurt Brassington[1], Aleksandar Dobric[1], Simone N. De Luca[1], Huei Jiunn Seow[1], Stavros Selemidis[1], Steven Bozinovski[1], Ross Vlahos[1]*

[1]School of Health and Biomedical Sciences, RMIT University, BUNDOORA, VIC 3083, Australia.

*** Correspondence:**
Professor Ross Vlahos, PhD
School of Health and Biomedical Sciences
RMIT University, PO Box 71, Bundoora, VIC 3083 Australia
Tel: +61 3 9925 7362
Email: ross.vlahos@rmit.edu.au

Keywords: viral exacerbation, COPD, muscle weakness, fiber type transformation, lung-to-muscle axis, myogenic disruption, conditioned medium.

Supplementary Material

1 SUPPLEMENTARY FIGURES

(A)

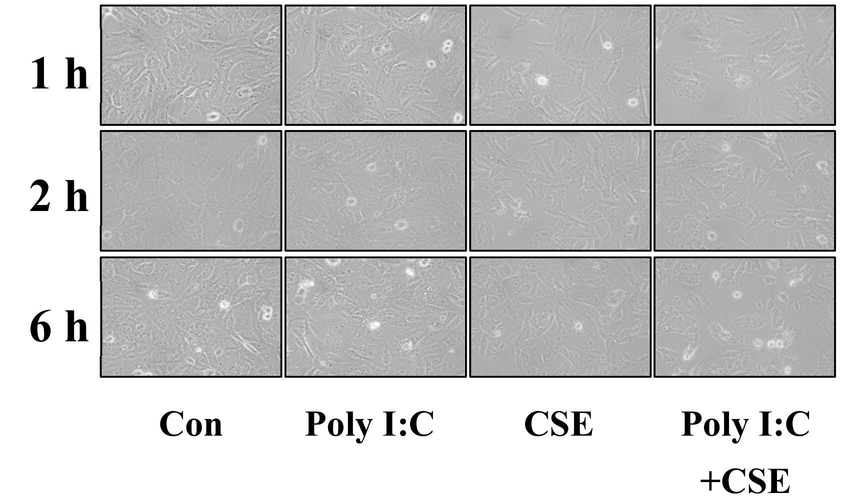

(B)

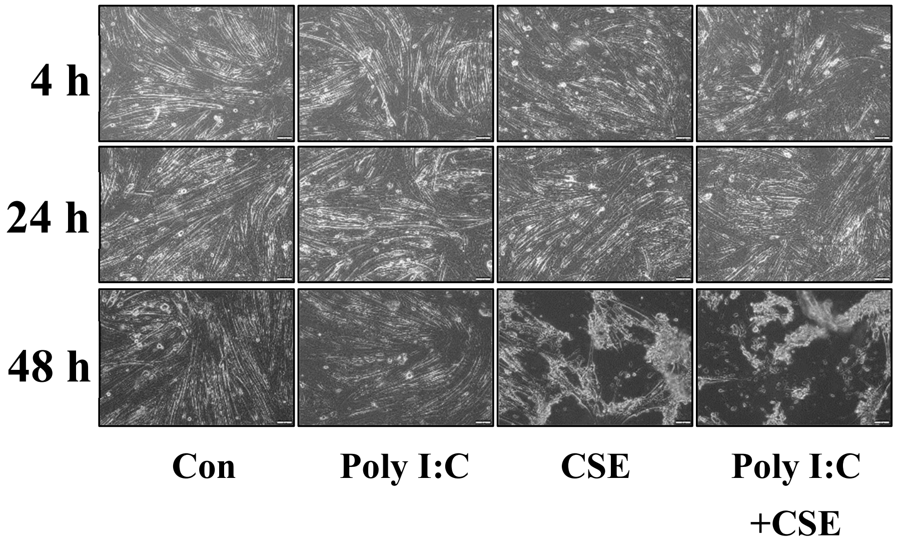

Supplementary Figure 1. Representative images of cell morphology/viability following (A) direct exposure of Poly I:C (TLR3 agonist), cigarette smoke extract (CSE) or their combination in BEAS−2B cells, or (B) C2C12 myotubes exposed to conditioned media derived from the respective conditions in BEAS−2B.
